# Association between vitamin D metabolites, vitamin D binding protein, and proteinuria in dogs

**DOI:** 10.1111/jvim.15912

**Published:** 2020-10-07

**Authors:** Matthew S. Miller, Adam J. Rudinsky, Brett G. Klamer, Dennis J. Chew, Valerie J. Parker

**Affiliations:** ^1^ Department of Veterinary Clinical Sciences The Ohio State University College of Veterinary Medicine Columbus Ohio USA; ^2^ Center for Biostatistics, Department of Biomedical Informatics The Ohio State University Columbus Ohio USA

**Keywords:** 25‐hydroxyvitamin D, calcitriol, kidney disease, urine protein:creatinine

## Abstract

**Background:**

Proteinuria has been associated with progression of renal disease and increased morbidity and mortality in dogs and people. In people, proteinuria also has been associated with hypovitaminosis D. Little is known about the relationship between vitamin D metabolism and proteinuria in dogs.

**Objectives:**

To further elucidate vitamin D status in dogs with protein‐losing nephropathy (PLN) and minimal to no azotemia. We hypothesized that vitamin D metabolites would be lower in dogs with PLN compared to healthy dogs.

**Animals:**

Twenty‐three client‐owned adult dogs with PLN and 10 healthy control dogs.

**Methods:**

Serum 25‐hydroxyvitamin D (25[OH]D), 1,25‐dihydroxyvitamin D (1,25[OH]_2_D), 24,25‐dihydroxyvitamin D (24,25[OH]_2_D), serum vitamin D binding protein (VDBP), and urine 25(OH)D concentrations were measured.

**Results:**

Compared to healthy dogs, dogs with PLN had lower concentrations of all vitamin D metabolites (*P* < .01). Correlations (rho; 95% confidence interval [CI]) in dogs with PLN are reported. Serum 25(OH)D and 24,25(OH)_2_D concentrations were positively correlated with albumin (*r* = 0.47; 0.07‐0.74), and 24,25(OH)_2_D was negatively correlated with urine protein‐to‐creatinine ratio (UPC; *r* = −0.54; −0.78 to −0.16). Urine 25(OH)D‐to‐creatinine ratio was negatively correlated with serum albumin concentration (*r* = −0.77; −0.91 to −0.50) and positively correlated with UPC (*r* = 0.79; 0.53‐0.91). Serum VDBP concentration was positively correlated with serum albumin concentration (*r* = 0.53; 0.05‐0.81).

**Conclusions and Clinical Importance:**

Dogs with PLN have decreased serum concentrations of vitamin D metabolites. Urine 25(OH)D‐to‐creatinine ratio and UPC are correlated in PLN dogs. Future studies are needed to assess additional management strategies for dogs with PLN.

**Abbreviations**
BCS: body condition score
CKD: chronic kidney disease
MCS: muscle condition score
PLN: protein‐losing nephropathy
UPC: urine protein:creatinine
VDBP: vitamin D binding protein
VDR: vitamin D receptor

## 1 INTRODUCTION

Chronic kidney disease (CKD) is commonly diagnosed in dogs, with a prevalence of up to 25% of dogs presented to veterinary teaching hospitals.^1^, ^2^, ^3^ Major consequences of CKD in dogs include development of proteinuria and hypovitaminosis D.^4^, ^5^ In people, proteinuria and hypovitaminosis D, characterized by decreased serum 25‐hydroxyvitamin D (25[OH]D) concentrations, are associated with progression of kidney disease and decreased survival,^6^, ^7^ and vitamin D status is inversely associated with magnitude of proteinuria.^8^, ^9^, ^10^, ^11^ Proteinuria in people is an independent predictor of vitamin D status in the absence of other causes of vitamin D dysregulation associated with kidney disease.^12^, ^13^

Similarly in dogs, proteinuria has been shown to be associated with progression of kidney disease and increased morbidity and death.^14^ An association between serum vitamin D concentration and proteinuria also has been identified in dogs. In a study of 19 dogs with azotemic CKD, serum 25(OH)D concentrations were inversely related to urine protein:creatinine (UPC) ratios.^15^ However, because of the complex pathophysiology of CKD‐mineral and bone disorder (CKD‐MBD), it was not possible to determine whether an independent association between proteinuria and vitamin D status existed separately from other CKD variables (eg, advanced azotemia). As a result, the association between proteinuria and vitamin D status in dogs with minimal to no azotemia remains unknown.

Our primary aim was to evaluate the relationship between vitamin D metabolites (25[OH]D, 1,25[OH]_2_D, and 24,25[OH]_2_D) and proteinuria assessed by UPC in dogs with protein‐losing nephropathy (PLN) and minimal to no azotemia. Our hypothesis was that, compared to healthy control dogs, vitamin D metabolites would be decreased in dogs with PLN, and vitamin D status (assessed by 25[OH]D) would be correlated with UPC.

## 2 MATERIALS AND METHODS

### 2.1 Case selection criteria

Client‐owned dogs prospectively recruited from the patient population referred to The Ohio State University Veterinary Medical Center (OSU‐VMC) between January 2014 and July 2015 for 2 other studies were included in the study.^4^, ^16^ Dogs were eligible for inclusion in the study if they were classified as having persistent proteinuria (ie, UPC > 0.5) and serum creatinine concentration <1.4 mg/dL, based on International Renal Interest Society (IRIS) CKD staging guidelines on at least 2 occasions. Causes of prerenal and postrenal proteinuria such as hyperadrenocorticism, cystoliths, or urinary neoplasia were excluded to the best of the attending clinicians' abilities based on a combination of clinical signs, physical examination findings, abdominal ultrasound findings, and urine culture. All included dogs had negative urine cultures. Abdominal ultrasound examination was performed in most dogs (n = 14).

Dogs were excluded from the study if they were <1 year of age, receiving medications known to affect proteinuria (eg, corticosteroids), had a serum creatinine concentration ≥1.4 mg/dL) or had clinical signs, physical examination findings, or abdominal imaging abnormalities consistent with concurrent diseases that would affect proteinuria. Dogs enrolled as controls were deemed healthy on the basis of a normal physical examination, CBC, serum biochemistry profile, and urinalysis. All owners signed a consent form before dogs were enrolled.^4^, ^16^

### 2.2 Study design

Each dog had a complete physical examination performed, including body weight, body condition score (BCS), and muscle condition score (MCS).^17^ All BCS (using the 9‐point scoring system) and MCS scores were assigned by 1 author (Valerie J. Parker). Blood pressure was measured using a Doppler ultrasonographic device and appropriately sized cuffs based on dog size. Blood was collected by jugular venipuncture for CBC and serum biochemistry. Urine was collected by cystocentesis for urinalysis, urine culture, and UPC. Additional serum and urine was stored at −80°C for analysis of vitamin D metabolites and vitamin D binding protein (VDBP). Information regarding medications, diets, and dietary supplements was recorded.

### 2.3 Vitamin D metabolites and VDBP analysis

Serum 25(OH)D and 1,25(OH)_2_D concentrations were measured by radioimmunoassay (RIA) and 24,25(OH)_2_D concentration was measured by liquid chromatography‐mass spectrometry (LC‐MS). Urine 25(OH)D concentration was measured by RIA and serum VDBP concentration was measured by ELISA. As has been reported elsewhere, urine 25(OH)D concentration was assessed relative to urine creatinine concentration.^18^ All vitamin D measurements were performed by a Vitamin D External Quality Assessment Scheme (DEQUAS)‐certified laboratory (Heartland Assays, Inc, Ames, Iowa).

### 2.4 Data analysis

Statistical analysis was performed using R 3.6.1 (R Core Team [2019], Vienna, Austria). Descriptive statistics were summarized using frequency and percentages for categorical variables and median and range for numerical variables. Spearman's rank correlation coefficients were calculated to determine correlation between 2 numerical variables. Wilcoxon rank sum tests and Fisher's exact test were utilized to compare average values and categorical associations between PLN and control dogs, respectively. Multivariable linear regression models were used to determine the association between serum vitamin D concentrations and other clinical variables, including serum creatinine concentration (mg/dL), serum albumin concentration (g/dL), UPC (mg/mg), age (years), and body weight (kg). In the final multivariable linear regression model and only in dogs with PLN, serum albumin concentration was used instead of UPC because the collinearity of these variables prevented both from being utilized in the final model. Serum concentrations of vitamin D metabolites and UPC were natural log transformed before fitting the multivariable regression models to assist with interpretation and to satisfy the assumptions required by linear regression. *P*‐values ≤.05 were considered significant and were not adjusted for multiplicity.

## 3 RESULTS

Twenty‐three dogs with PLN and 10 control dogs were included. Median age for dogs with PLN was 9.9 years (range, 3.1‐13.5 years). Median age for control dogs was 4.3 years (range, 1.4‐10.3 years). Dogs with proteinuria were significantly older than control dogs (*P* < .001). Breeds represented among dogs with PLN were Yorkshire Terrier (n = 3), Cocker Spaniel (n = 2), Fox Terrier (n = 2), Labrador Retriever (n = 2), Miniature Schnauzer (n = 2), and mixed breed (n = 2). There were 1 each of the following breeds: Akita, Australian Shepherd, Bichon Frise, Chihuahua, Cavalier King Charles Spaniel, Doberman Pinscher, Golden Retriever, Soft Coated Wheaten Terrier, Shetland Sheep dog, and Welsh Terrier. Seventeen spayed female and 6 castrated male dogs were included. Control dogs included mixed breed (n = 4), American pit Bull Terrier (n = 3), German Shepherd (n = 2), and Rottweiler (n = 1). Six dogs were castrated males and 4 were spayed females.

Median body weight of dogs with PLN was 10.2 kg (range, 2.0‐43.0 kg). Using the 9‐point scoring system, median BCS was 7 (range, 4‐9). Four dogs had an ideal BCS (4, 5), and 19 dogs were overconditioned (BCS > 5), with 6/19 overconditioned dogs characterized as obese (BCS 8‐9). The MCS was assessed to be normal in 17 dogs. Muscle loss was noted to be mild in 4 dogs, and moderate in 2 dogs. Median body weight of control dogs was 26.2 kg (range, 13.5‐47.0 kg). Median BCS was 6 (range, 4.5‐8). All control dogs had normal MCS.

**FIGURE 1:**
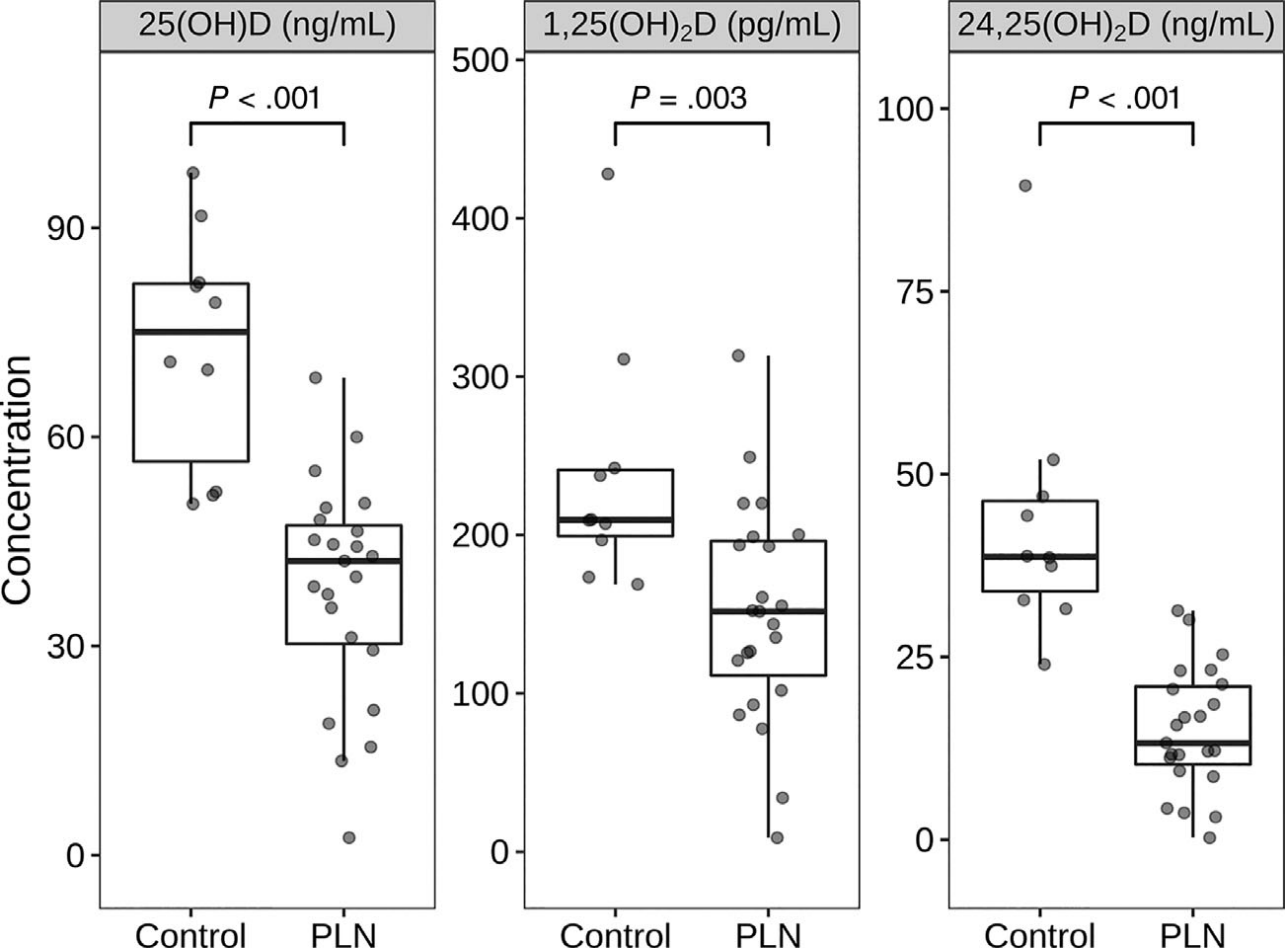
Serum vitamin D concentrations in dogs with PLN and control dogs. Each dot represents a dog. The boxes represent the 25th and 75th percentiles, and the central lines in the boxes represent the median (50th percentile) values. The whiskers extend up to 1.5*IQR below and above the 25th and 75th percentiles respectively. Points above and below the whiskers are indications for outlier values. The dogs with protein‐losing nephropathy (PLN) had lower vitamin D concentrations (Wilcoxon rank‐sum test, *P* < .001)

**FIGURE 2:**
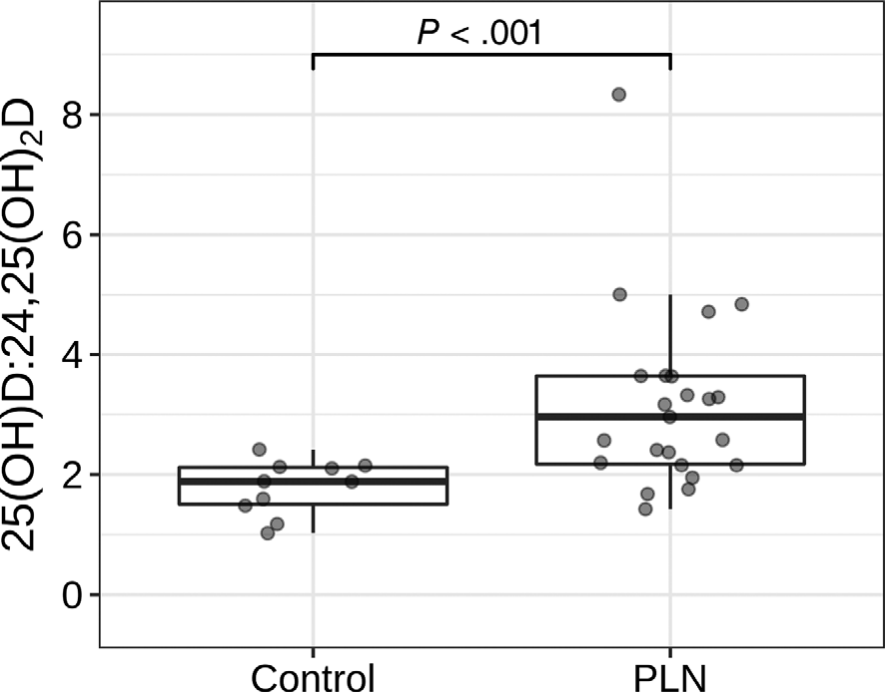
Serum 25(OH)D to 24,25(OH)_2_D ratios in dogs with protein‐losing nephropathy (PLN) and control dogs. Each dot represents a dog. The boxes represent the 25th and 75th percentiles, and the central lines in the boxes represent the median (50th percentile) values. The whiskers extend up to 1.5*IQR below and above the 25th and 75th percentiles respectively. Points above and below the whiskers are indications for outlier values. The dogs with PLN had higher average 25(OH)D to 24,25(OH)_2_D ratios (Wilcoxon rank‐sum test, *P* < .001)

Laboratory variables, vitamin D metabolite concentrations, and vitamin D metabolite ratios from the PLN and control cohorts are presented in Table 1. Median UPC of the PLN cohort was 4.8 (range, 1.7‐27.5). Median blood pressure was 160 mm Hg (range, 120‐240 mm Hg). None of the control dogs was proteinuric. One dog had an increased systolic blood pressure of 180 mm Hg, but no specific underlying etiology was identified to account for this dog's hypertension. Dogs with PLN had significantly lower serum albumin concentrations (*P* = .02) and urine specific gravity (*P* < .001) and significantly higher UPC (*P* < .001) and blood pressure (*P* = .03) compared to control dogs. Dogs with PLN had significantly lower serum 25(OH)D (*P* < .001), 1,25(OH)_2_D (*P* = .003), and 24,25(OH)_2_D (*P* < .001) concentrations compared to controls (Figure 1). Dogs with PLN had significantly higher average 25(OH)D‐to‐24,25(OH)_2_D ratio compared to control dogs (*P* > .001), with no significant difference in average 1,25(OH)_2_D‐to‐25(OH)D ratio noted between groups (Figure 2).

**TABLE 1:** Laboratory variables and serum vitamin D metabolite concentrations of dogs with PLN and healthy control dogs. Results are presented as median (range). Results with *P*‐values <.05 are bolded

Variable (reference range)	Dogs with PLN (n = 23)	Control dogs (n = 10)	*P*‐value
Age (years)	**9.9 (3.0‐13.5)**	**4.3 (1.4‐10.3)**	**<.001**
Sex			
Female	4 (40%)	17 (74%)	.11
Male	6 (60%)	6 (26%)	
BCS	7 (4‐9)	6 (4.5‐8)	.07
Body weight (kg)	**10.2 (2‐43)**	**26.2 (13.5‐47)**	**.01**
Hematocrit (37%‐56%)	47 (30‐62)	51 (45‐55)	.18
BUN (5‐20 mg/dL)	18 (6‐58)	18.5 (14‐23)	1.0
Creatinine (0.6‐1.6 mg/dL)	**0.7 (0.5‐1.2)**	**1.0 (0.8‐1.2)**	**.006**
Phosphorus (3.2‐8.1 mg/dL)	3.9 (2.7‐8.1)	4.0 (2.7‐5.6)	.89
Albumin (2.9‐4.2 g/dL)	**3.1 (0.9‐4.1)**	**3.7 (3.0‐4.0)**	**.01**
USG	**1.020 (1.007‐1.065)**	**1.043 (1.031‐1.053)**	**<.001**
UPC	**4.8 (1.7‐27.5)**	**0.1 (0.1‐0.1)**	**<.001**
BP (mm Hg)	**160 (120‐240)**	**137 (100‐180)**	**.03**
Serum 25(OH)D (ng/mL)	**42.2 (2.5‐68.5)**	**75.1 (50.4‐97.9)**	**<.001**
Serum 1,25(OH)_2_D (pg/mL)	**151.8 (8.9‐313.3)**	**209.6 (168.9‐428)**	**.003**
Serum 24,25(OH)_2_D (ng/mL)	**13.2 (0.3‐31.3)**	**38.7 (24‐89.5)**	**<.001**
Urine 25(OH)D (pg/mL) (n = 20)	1630.25 (475.7‐11 635.0)	N/A	
Urine 25(OH)D: creatinine ratio (pg/mg) (n = 20)	28.44 (5.48‐181.62)	N/A	
Serum VDBP (μg/mL)	200.45 (97.6‐541.9) (n = 16)	146.1 (95.6‐305.4) (n = 9)	.10
1,25(OH)_2_D:25(OH)D (pg/ng)	3.68 (1.56‐9.38)	3.21 (2.06‐4.67)	.29
25(OH)D:24,25(OH)_2_D	**2.96 (1.42‐8.33)**	**1.89 (1.02‐2.42)**	**<.001**

Abbreviations: BCS, body condition score; BUN, blood urea nitrogen; PLN, protein‐losing nephropathy; UPC, urine protein‐to‐creatinine ratio; USG, urine‐specific gravity; VDBP, vitamin D binding protein.

Of the 23 dogs with PLN, urine 25(OH)D and serum VDBP concentrations were available for 20 and 16 dogs, respectively. Serum VDBP concentrations were available in 9 control dogs. No significant difference was found in serum VDBP concentrations between control dogs and dogs with PLN. Urine 25(OH)D was not measured in control dogs because of lack of availability of extra samples.

**FIGURE 3:**
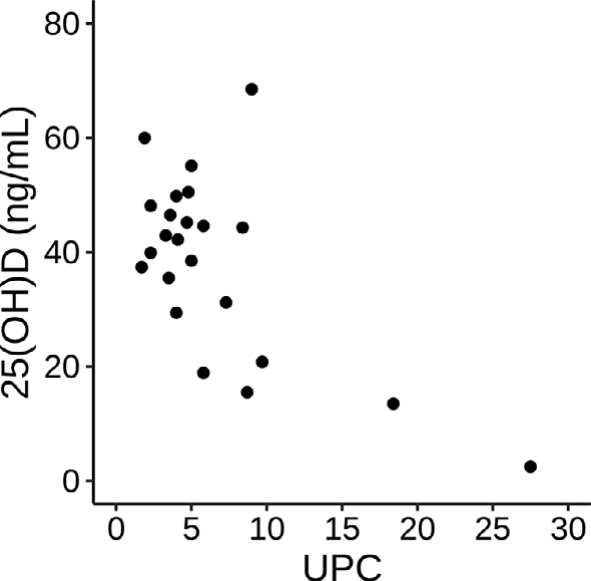
Scatterplot of 25(OH)D and urine protein:creatinine ratio (UPC) in dogs with protein‐losing nephropathy (PLN). Spearman's rho of −0.40 (95% CI: −0.69, −0.02; *P* = .06)

**FIGURE 4:**
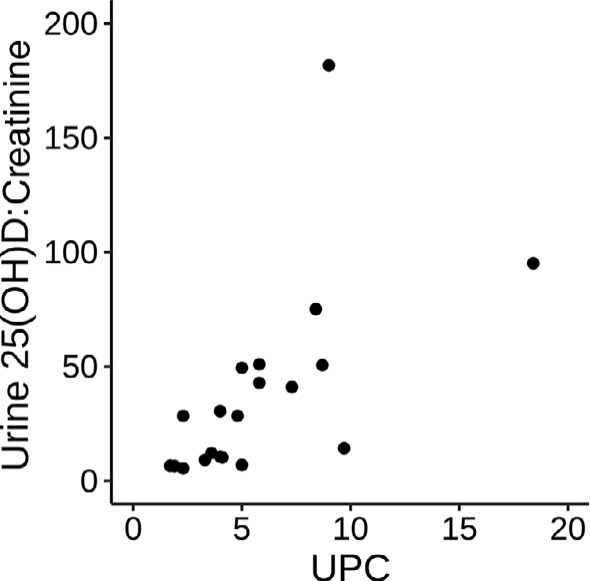
Scatterplot of urine 25(OH)D:creatinine and urine protein:creatinine ratio (UPC) in dogs with protein‐losing nephropathy (PLN). Spearman's rho of 0.78 (95% CI: 0.91, 0.53; *P* < .001)

Spearman correlations between vitamin D metabolites and laboratory variables in dogs with PLN are presented in Table 2. Serum 25(OH)D concentrations were significantly negatively correlated with serum creatinine concentration (*P* = .01) and positively correlated with serum albumin concentration (*P* = .02). Serum 25(OH)D concentration was not significantly correlated with UPC (*P* = .06; Figure 3). Serum 24,25(OH)_2_D concentrations were significantly negatively correlated with serum creatinine concentration (*P* = .009), serum phosphorus concentration (*P* = .003), and UPC (*P* = .008), and were positively correlated with serum albumin concentration (*P* = .02) and body weight (*P* = .05). No significant association was found between any vitamin D metabolite and blood pressure. Serum 1,25(OH)_2_D concentration had no significant correlations to any variable. Urine 25(OH)D‐to‐creatinine ratios were positively correlated with UPC (*P* < .001; Figure 4) and negatively correlated with serum albumin concentration (*P* < .001). Serum VDBP concentrations were positively correlated with serum albumin concentration (*P =* .03) and age (*P =* .01;Table 3).

**TABLE 2:** Spearman correlations (rho) between serum vitamin D metabolites and variables in dogs with PLN. Statistically significant correlations are bolded

	25(OH)D		1,25(OH)_2_D		24,25(OH)_2_D	
Variable	Rho	*P*‐value	Rho	*P*‐value	Rho	*P*‐value
Age	0.22	.31	0.14	.52	0.34	.11
BCS	−0.11	.14	−0.08	.11	−0.35	.75
Body weight	**0.54**	**.007**	0.17	.43	**0.42**	**.05**
Hematocrit	0.03	.88	0.29	.18	0.02	.91
BUN	−0.19	.38	−0.13	.57	−0.33	.13
Creatinine	**−0.51**	**.01**	−0.11	.62	**−0.53**	**.009**
Phosphorus	−0.16	.46	−0.38	.07	**−0.59**	**.003**
Albumin	**0.47**	**.02**	0.27	.22	**0.47**	**.02**
USG	−0.22	.31	−0.18	.40	−0.27	.21
UPC	−0.40	.06	−0.12	.58	**−0.54**	**.008**
Blood pressure	−0.09	.69	−0.09	.69	−0.12	.59
Serum VDBP	0.41	.11	0.34	.20	0.42	.11

Abbreviations: BCS, body condition score; BUN, blood urea nitrogen; PLN, protein‐losing nephropathy; UPC, urine protein‐to‐creatinine ratio; USG, urine‐specific gravity; VDBP, vitamin D binding protein.

**TABLE 3:** Spearman correlations (rho) between urine 25(OH)D, serum VDBP, and variables in dogs with PLN

	Urine 25(OH)D:creatinine		Serum VDBP	
Variable	Rho	*P*‐value	Rho	*P*‐value
USG	0.2	.39	−0.38	.14
UPC	**0.79**	**<.001**	−0.48	.06
Albumin	**−0.77**	**<.001**	**0.53**	**.03**
Phosphorus	0.15	.53	−0.28	.29
Age	−0.2	.39	**0.61**	**.01**

The bolded values are statistically significant (ie, *P* value ≤ 0.05). Abbreviations: PLN, protein‐losing nephropathy; UPC, urine protein‐to‐creatinine ratio; USG, urine‐specific gravity; VDBP, vitamin D binding protein.

To explore the association of the combination of age, body weight, UPC, serum albumin concentration, and serum creatinine concentration on the vitamin D metabolites, we used multivariable linear regression models. As seen in Table 4, we found evidence for an association of each metabolite with UPC when evaluating all 33 dogs (23 PLN dogs and 10 control dogs). For every 10% increase in UPC, there was an estimated 2.6%, 2.0%, and 4.3% decrease in 25(OH)D, 1,25(OH)_2_D, and 24,25(OH)_2_D, respectively. A significant association also was found between 24,25(OH)_2_D and age. A separate analysis was performed to determine the association of serum creatinine and albumin concentrations on vitamin D metabolites in the PLN group. As seen in Table 5, an association was found between serum albumin concentration and all vitamin D metabolites, whereas both 24,25(OH)_2_D and 25(OH)D were associated with serum creatinine concentration. For every 1 g/dL increase in serum albumin concentration, there was an estimated 62.5%, 39.9%, and 60.1% increase in serum 25(OH)D, 1,25(OH)_2_D, and 24,25(OH)_2_D concentrations, respectively.

**TABLE 4:** Multivariable regression evaluating effect of urine protein‐to‐creatinine ratio (UPC), adjusting for age and weight, on vitamin D metabolites

	Log 1,25(OH)_2_D			Log 24,25(OH)_2_D			Log 25(OH)D		
Predictor	Est.	CI	*P*‐value	Est.	CI	*P*‐value	Est.	CI	*P*‐value
(Intercept)	4.57	3.80‐5.33	**<.001**	1.85	1.00‐2.70	**<.001**	3.1	2.47‐3.73	**<.001**
Log UPC	−0.21	−0.36 to 0.07	**.005**	−0.46	−0.62 to ‐0.3	**<.001**	−0.27	−0.39 to −0.15	**<.001**
Age	0.05	−0.04 to 0.13	.19	0.11	0.02 to 0.20	**.01**	0.06	−0.0002 to 0.13	.05
Weight (kg)	0.01	−0.01 to 0.03	.43	0.02	−0.01 to 0.04	.14	0.01	−0.003 to 0.03	.12
N	33			33			33		
R^2^/R^2^ adjusted	0.313/0.242			0.630/0.592			0.539/0.491		

The bolded values are statistically significant (ie,*P* value ≤ 0.05).

**TABLE 5:** Multivariable regression evaluating effects of creatinine and albumin, adjusting for age and weight, on vitamin D metabolites in dogs with protein‐losing nephropathy (PLN)

	Log 1,25(OH)_2_D			Log 24,25(OH)_2_D			Log 25(OH)D		
Predictor	Est.	CI	*P*‐value	Est.	CI	*P*‐value	Est.	CI	*P*‐value
(Intercept)	3.69	1.78 to 5.61	**.001**	1.57	−0.19 to 3.32	.08	3.13	1.86 to 4.40	**<.001**
Creatinine (mg/dL)	−0.63	−1.98 to 0.73	.35	−2.14	−3.39 to −0.9	**.002**	−1.56	−2.46 to −0.66	**.002**
Albumin (g/dL)	0.49	0.06‐0.91	**.03**	0.47	0.08‐0.86	**.02**	0.34	0.05‐0.62	**.02**
Age	0.01	−0.1 to 0.12	.87	0.08	−0.02 to 0.19	.10	0.04	−0.04 to0.11	.33
Weight (kg)	0.01	−0.02 to 0.03	.62	0.02	−0.002 to 0.04	.07	0.01	−0.001 to 0.03	.07
N	23			23			23		
R^2^/R^2^ adjusted	0.438/0.314			0.741/0.684			0.723/0.661		

The bolded values are statistically significant (ie,*P* value ≤ 0.05).

The dogs with PLN were eating commercial diets with a wide range of cholecalciferol concentrations (10‐91 International Units [IU] per 100 kcal). Two dogs were eating a diet specifically designed for dogs with kidney disease. Many dogs were receiving supplemental foods for people as treats. Total daily dietary cholecalciferol intake could not be determined. Dogs with PLN were receiving the following medications: enalapril (n = 2), amlodipine (n = 1), diethylstilbestrol (n = 1), doxycycline (n = 1), phenylpropanolamine (n = 1), and tramadol (n = 1). Concurrent disease processes included: uncontrolled hypertension (n = 3), liver nodules (n = 3), historical complete or partial cruciate ligament rupture (n = 3), known or presumptive osteoarthritis (n = 2), allergic skin disease (n = 2), C6‐T2 myelopathy (n = 1), suspected systemic lupoid onychodystrophy (n = 1), pododermatitis (n = 1), previously excised grade II mast cell tumor (n = 1), diabetes mellitus (n = 1), tracheal collapse (n = 1), completely excised high‐low grade oral fibrosarcoma (n = 1), splenic nodules (n = 1), and keratoconjunctivitis sicca (n = 1).

The control dogs all received complete and balanced commercial adult maintenance diets. Aside from 1 control dog on diphenhydramine, the control dogs were not receiving any medications except monthly flea or heartworm preventatives.

## 4 DISCUSSION

We found that proteinuria in minimally to non‐azotemic dogs is associated with decreased serum concentrations of vitamin D metabolites compared to control dogs. Serum 25(OH)D and 24,25(OH)_2_D concentrations showed significant univariable correlations with body weight and serum creatinine and albumin concentrations, whereas 24,25(OH)_2_D concentrations had significant univariable correlations with serum phosphorus concentrations and UPC. No significant univariable correlations were detected between serum 1,25(OH)_2_D concentration and any laboratory variable. Urine 25(OH)D‐to‐creatinine ratios had significant univariable correlations with serum albumin concentration and UPC whereas serum VDBP concentration had significant univariable correlations with serum albumin concentration and age. Multivariable linear regression showed a significant, covariate‐adjusted effect of UPC among all dogs, and serum albumin concentration among PLN dogs on all vitamin D metabolites, whereas serum creatinine concentration had a significant, covariate‐adjusted effect on both serum 25(OH)D and 24,25(OH)_2_D concentrations among PLN dogs.

Kidney disease can affect the development of hypovitaminosis D in several ways, as has been previously described. Conversely, hypovitaminosis D itself can influence the development and progression of proteinuria.^19^, ^20^, ^21^, ^22^ Podocytes express both 1α‐hydroxylase, the enzyme responsible for conversion of 25(OH)D to 1,25[OH]_2_D (calcitriol), and the vitamin D receptor (VDR). Calcitriol and other VDR analogues regulate proteins in the slit diaphragm (eg, nephrin, podocin) that are responsible for maintaining a functional glomerular basement membrane.^19^, ^20^, ^21^, ^22^ Activation of the VDR suppresses renin, has anti‐fibrotic effects, and decreases glomerulosclerosis.^22^, ^23^, ^24^

In people, several studies have demonstrated similar associations between vitamin D metabolites and proteinuria or albuminuria.^25^, ^26^, ^27^, ^28^ One study identified patients with focal segmental glomerulosclerosis, a disorder associated with proteinuria, to be at risk of vitamin D deficiency.^29^ Another study in children with nephrotic syndrome and normal glomerular filtration rate showed 25(OH)D deficiency in all patients, but serum 1,25(OH)_2_D concentrations were normal in most patients.^30^ A multifactorial explanation likely exists for the hypovitaminosis D in these diseases in people and in our study of dogs with PLN, including decreased nutritional intake, decreased synthesis of active vitamin D, and the potential for loss of bound vitamin D as a consequence of VDBP loss.

Decreased nutritional intake of cholecalciferol has been hypothesized to influence 25(OH)D status in dogs with a variety of diseases, including protein‐losing enteropathy (PLE) and CKD. An association has been reported between decreased vitamin D intake and vitamin D deficiency in people.^31^, ^32^ Dogs in our study were eating diets with a wide range of cholecalciferol (10‐91 IU per 100 kcal), and they should have been receiving their minimum requirements. Previous studies have shown that 25(OH)D status cannot be reliably predicted by dietary intake.^33^, ^34^, ^35^ Altered intestinal absorption of cholecalciferol may affect 25(OH)D status^35^, ^36^ but no historical, physical examination, or diagnostic findings indicated clinically relevant gastrointestinal disease in the dogs in our study.

Alterations in enzymatic activity can affect serum vitamin D concentrations. Decreased transformation of cholecalciferol to 25(OH)D may result from altered 25‐hydroxylase activity.^37^ Additionally, the resultant decrease in nephron mass can lead to decreased expression of 1α‐hydroxylase, which can result in decreased conversion of 25(OH)D to calcitriol. We attempted to minimize this confounding factor by excluding dogs with serum creatinine concentrations ≥1.4 mg/dL. Dogs with IRIS stage 1 CKD did not have significantly different concentrations of any vitamin D metabolites compared to healthy controls.^4^ In our multivariable regression, serum creatinine concentration had an independent association with both 25(OH)D and 24,25(OH)_2_D, but not with 1,25(OH)_2_D. The model estimated decreases in these metabolites with increasing serum creatinine concentration. Therefore, an effect of creatinine could not be entirely eliminated from our study. Additionally, some breed‐specific reference ranges exist, and some of the dogs in our study may have had mild azotemia based on blood urea nitrogen concentrations.^38^

Ratios of 25(OH)D‐to‐24,25(OH)_2_D and 1,25(OH)_2_D‐to‐25(OH)D were calculated. The dogs with PLN had significantly higher 25(OH)D‐to‐24,25(OH)_2_D ratios compared to the control group. This ratio has been shown to be potentially useful as an indicator of hypovitaminosis D in people.^39^ In our study, this increase in the 25(OH)D‐to‐24,25(OH)_2_D ratio may indicate decreased conversion of 25(OH)D to 24,25(OH)_2_D, which would be expected in hypovitaminosis D. The lack of difference in 1,25(OH)_2_D‐to‐25(OH)D ratios between groups makes decreased ability to convert 25(OH)D to active 1,25(OH)_2_D less likely in our PLN dogs, seemingly further supporting alternative explanations for hypovitaminosis D, including urinary loss.

Urine loss of vitamin D is a potential contributor to decreased serum concentrations of vitamin D metabolites because of loss of either VDBP‐complexed or albumin‐complexed vitamin D metabolites. In people, approximately 85% to 90% of 25(OH)D is bound to VDBP, which is responsible for transporting 25(OH)D in blood, whereas 10% to 15% is bound to albumin.^40^ The VDBP‐25(OH)D complex normally is filtered by the glomerulus and then reabsorbed in the proximal tubule. This process is mediated by the membrane receptor megalin as well as cubilin and disabled‐2 (Dab2).^41^, ^42^ In a diseased kidney, or when there is decreased expression of megalin, cubilin or Dab2, decreased reabsorption at the tubular brush border may result in increased loss of the VDBP‐25(OH)D complex and subsequent increased urinary excretion of 25(OH)D.^43^, ^44^ Vitamin D metabolites complexed to albumin may be able to bypass the megalin/cubilin system entirely. Although loss of vitamin D metabolites complexed to VDBP is likely the more important source of loss, albumin loss also likely contributed to some extent.

Despite lacking urine VDBP data in control dogs, the significant negative correlation of 24,25(OH)_2_D and positive correlation of urine 25(OH)D with UPC, as well as the significant negative correlation of urine 25(OH)D with albumin seen in our study may further support the hypothesis of increased urinary loss of vitamin D in patients with proteinuria. Although PLN dogs had no significant difference in serum VDBP concentration compared to control dogs, some studies have shown a compensatory increase in serum VDBP concentration in people with CKD and increased urinary VDBP loss.^45^ Additionally, using multivariable linear regression, all vitamin D metabolites were shown to be independently affected by UPC, further supporting this hypothesis.

Conflicting information is found in human medical literature about the relevance of this loss. Increased urinary loss of 25(OH)D may be a major cause of hypovitaminosis D in people with nephrotic syndrome both because of decreased reabsorption of 25(OH)D into the blood as well as decreased conversion of 25(OH)D to 1,25(OH)_2_D.^46^, ^47^, ^48^ However, a recent report in people failed to show a difference in serum 25(OH)D and 1,25(OH)_2_D concentrations despite decreased urinary loss of VDBP with anti‐proteinuric treatment.^44^ In rats, urinary VDBP was shown to potentially be a biomarker for tubulointerstitial disease.^49^ A recent study evaluated the use of urinary VDBP as a biomarker of renal tubular injury in dogs.^50^ In that study, no urinary VDBP was detected in healthy dog urine, a but CKD dogs did have VDBP urinary loss that increased with stage of CKD, even without proteinuria.

In our study, serum VDBP concentration was not significantly different between dogs with PLN and control dogs. There are conflicting reports of finding either increased^45^ or decreased^44^ serum or plasma VDBP concentrations in people with CKD, despite increased urinary loss of VDBP. Serum VDBP concentrations have not been evaluated in people with nephrotic syndrome. Serum VDBP concentration is negatively associated with age in people^40^, ^45^ but it was positively correlated with age in our dogs with PLN (*r* = 0.61; *P* = .01). The significance of this observation is unknown. The significant positive correlation of serum albumin and VDBP concentrations may further support an effect of protein loss on VDBP.

It would have been ideal to measure urinary VDBP in our dogs, but we did not have enough urine saved to do so. More work is needed to determine the utility of measurement of urine vitamin D metabolites and VDBP. It is also unknown if it is more appropriate to report urine 25(OH)D or VDBP as concentrations or as a ratio compared to urine creatinine concentration. In a study of 99 people with CKD, urinary VDBP excretion (nmol per 24 hours) closely correlated with urinary VDBP concentration determined in the second voided urine of the morning (nmol/L) as well as the urinary VDBP‐to‐creatinine ratio in the second voided urine of the morning (nmol/mmol).^51^

Significant positive correlations were observed between serum albumin and both 25(OH)D and 24,25(OH)_2_D concentrations. In people with CKD, those with lower serum 25(OH)D concentrations also were more likely to have hypoalbuminemia.^52^ A complex relationship likely exists between these variables. Hypoalbuminemia can result from proteinuria, inflammation, and malnutrition.^53^, ^54^, ^55^ Decreased vitamin D status previously has been correlated with hypoalbuminemia in dogs with chronic enteropathies.^36^, ^56^ Loss of albumin complexed with 25(OH)D partially may explain this finding, but given that only about 10% to 15% of 25(OH)D is bound to albumin in the circulation, it seems more likely that it is simply a reflection of general protein loss, including VDBP and its complexed vitamin D metabolites, rather than a causal link between albumin loss and hypovitaminosis D. Low vitamin D status also could affect albumin by its effects on inflammation.^57^, ^58^ Thus, the lack of vitamin D's immunomodulation actually may result in hypoalbuminemia, independent of urinary loss of albumin.^59^ Although malnutrition often is cited as a predisposing factor to hypoalbuminemia, only 1 dog was reported to be underconditioned and 10 dogs were noted to have variable degrees of muscle atrophy, with most (n = 7) having only mild atrophy.

Our study had a few limitations. The control dog cohort included fewer dogs than the PLN cohort, and the dogs were not age‐matched. No difference in serum 25(OH)D concentrations by age however was found in a study of 320 dogs.^35^ Regardless, it is difficult to assess what effects age and body weight may have had on the variables evaluated. The number of PLN dogs also was low. This could have led to a decrease in statistical power, potentially explaining some of the borderline significant results (eg, correlation between 25(OH)D and UPC). Significant intraindividual variation can occur in day‐to‐day UPC in proteinuric dogs,^60^ especially depending on methods of analysis (single, averaged, or 3‐day pooled samples).^61^ Although no studies to our knowledge have evaluated intraindividual variation in serum 25(OH)D concentrations in dogs, people tend to have fairly consistent serum 25(OH)D concentrations in a given year.^62^, ^63^

A major limitation was the inability to compare urine 25(OH)D and serum VDBP concentrations to control dogs. This information would be useful in further supporting or refuting the idea that hypovitaminosis D in proteinuric patients is caused by loss of bound vitamin D metabolites in the urine. Future studies ideally should determine the combined effects of both CKD and PLN on vitamin D metabolites. An ideal study would include additional groups (eg, control, proteinuric nonazotemic, azotemic nonproteinuric, and azotemic proteinuric dogs).

In conclusion, vitamin D metabolites were significantly decreased in dogs with PLN compared to healthy control dogs. It is likely a multifactorial process involving altered glomerular basement membrane function, decreased synthesis of calcitriol, systemic inflammation, and loss of vitamin D metabolites in urine. Although vitamin D supplementation has been shown to decrease proteinuria in people,^64^, ^65^ it has yet to be established whether supplementation with any form of vitamin D (cholecalciferol, ergocalciferol) or vitamin D metabolite (25[OH]_2_D, calcitriol, or calcitriol analogue) can improve proteinuria, slow disease progression, or decrease mortality in dogs with PLN.

## CONFLICT OF INTEREST DECLARATION

Authors declare no conflict of interest.

## OFF‐LABEL ANTIMICROBIAL DECLARATION

Authors declare no off‐label use of antimicrobials.

## INSTITUTIONAL ANIMAL CARE AND USE COMMITTEE (IACUC) OR OTHER APPROVAL DECLARATION

The Ohio State University IACUC approval.

## HUMAN ETHICS APPROVAL DECLARATION

Authors declare human ethics approval was not needed for this study.

## Supporting information


**Appendix** S1: Supporting Information

## References

[1] Bartlett PC , Van Buren JW , Neterer M , et al. Disease surveillance and referral bias in the veterinary medical database. Prev Vet Med. 2010;94:264‐271. 20129684 10.1016/j.prevetmed.2010.01.007

[2] Lund EM , Armstrong PJ , Kirk CA , Kolar LM , Klausner JS . Health status and population characteristics of dogs and cats examined at private veterinary practices in the United States. J Am Vet Med Assoc. 1999;214:1336‐1341. 10319174

[3] Guidi G , Rossini C , Cinelli C , et al. Canine chronic kidney disease: retrospective study of a 10‐year period of clinical activity In: Pugliese A, Gaiti A, Boiti C, eds. Veterinary Science: Current Aspects in Biology, Animal Pathology, Clinic and Food Hygiene. New York, NY: Springer; 2012:115‐118.4.

[4] Parker VJ , Harjes LM , Dembek K , Young GS , Chew DJ , Toribio RE . Association of vitamin D metabolites with parathyroid hormone, fibroblast growth factor‐23, calcium, and phosphorus in dogs with various stages of chronic kidney disease. J Vet Intern Med. 2017;31:791‐798. 28186657 10.1111/jvim.14653 PMC5435068

[5] Wehner A , Hartmann K , Hirschberger J . Associations between proteinuria, systemic hypertension and glomerular filtration rate in dogs with renal and non‐renal diseases. Vet Rec. 2008;162:141‐147. 18245745 10.1136/vr.162.5.141

[6] Mehrotra R , Kermah DA , Salusky IB , et al. Chronic kidney disease, hypovitaminosis D, and mortality in the United States. Kidney Int. 2009;76:977‐983. 19657329 10.1038/ki.2009.288 PMC3791220

[7] Wolf M , Shah A , Gutierrez O , et al. Vitamin D levels and early mortality among incident hemodialysis patients. Kidney Int. 2007;72:1004‐1013. 17687259 10.1038/sj.ki.5002451

[8] Filipov JJ , Zlatkov BK , Dimitrov EP , Svinarov DA . Higher 25‐hydroxyvitamin D levels are associated with lower proteinuria in kidney transplant recipients. Exp Clin Transplant. 2016;14:629‐633. 27483020 10.6002/ect.2015.0344

[9] Shroff R , Aitkenhead H , Costa N , et al. Normal 25‐hydroxyvitamin D levels are associated with less proteinuria and attenuate renal failure progression in children with CKD. J Am Soc Nephrol. 2016;27:314‐322. 26069294 10.1681/ASN.2014090947 PMC4696567

[10] Cravedi P , Remuzzi G . Pathophysiology of proteinuria and its value as an outcome measure in chronic kidney disease. Br J Clin Pharmacol. 2013;76:516‐523. 23441592 10.1111/bcp.12104 PMC3791975

[11] Matsushita K , van der Velde M , Astor BC , et al. Association of estimated glomerular filtration rate and albuminuria with all‐cause and cardiovascular mortality in general population cohorts: a collaborative meta‐analysis. Lancet. 2010;375:2073‐2081. 20483451 10.1016/S0140-6736(10)60674-5 PMC3993088

[12] de Boer IH , Ioannou GN , Kestenbaum B , Brunzell JD , Weiss NS . 25‐hydroxyvitamin D levels and albuminuria in the third national health and nutrition examination survey (NHANES III). Am J Kidney Dis. 2007;50:69‐77. 17591526 10.1053/j.ajkd.2007.04.015

[13] Mehrotra R , Kermah D , Budoff M , et al. Hypovitaminosis D in chronic kidney disease. Clin J Am Soc Nephrol. 2008;3:1144‐1151. 18417740 10.2215/CJN.05781207 PMC2440286

[14] Jacob F , Polzin DJ , Osborne CA , et al. Evaluation of the association between initial proteinuria and morbidity rate or death in dogs with naturally occurring chronic renal failure. J Am Vet Med Assoc. 2005;226:393‐400. 15702689 10.2460/javma.2005.226.393

[15] Galler A , Tran JL , Krammer‐Lukas S , et al. Blood vitamin levels in dogs with chronic kidney disease. Vet J. 2012;192:226‐231. 21767966 10.1016/j.tvjl.2011.06.026

[16] Parker VJ , Fascetti AJ , Klamer BG . Amino acid status in dogs with protein‐losing nephropathy. J Vet Intern Med. 2019;33:680‐685. 30784117 10.1111/jvim.15436 PMC6430895

[17] Baldwin KBJ , Buffington T , Freeman LM , Grabow M , Legred J , Ostwald D . AAHA nutritional assessment guidelines for dogs and cats. J Am Anim Hosp Assoc. 2010;46:285‐296. 20610704 10.5326/0460285

[18] Anderson RL , Ternes SB , Strand KA , Rowling MJ . Vitamin D homeostasis is compromised due to increased urinary excretion of the 25‐hydroxycholecalciferol‐vitamin D‐binding protein complex in the Zucker diabetic fatty rat. Am J Phys Endocrine Met. 2010;299:E959‐E967. 10.1152/ajpendo.00218.2010 20876762

[19] Yamauchi K , Takano Y , Kasai A , et al. Screening and identification of substances that regulate nephrin gene expression using engineered reporter podocytes. Kidney Int. 2006;70:892‐900. 16820792 10.1038/sj.ki.5001625

[20] Trohatou O , Tsilibary E‐F , Charonis A , Iatrou C , Drossopoulou G . Vitamin D3 ameliorates podocyte injury through the nephrin signalling pathway. J Cell Mol Med. 2017;21:2599‐2609. 28664547 10.1111/jcmm.13180 PMC5618699

[21] Sonneveld R , Ferre S , Hoenderop JG , et al. Vitamin D down‐regulates TRPC6 expression in podocyte injury and proteinuric glomerular disease. Am J Pathol. 2013;182:1196‐1204. 23385000 10.1016/j.ajpath.2012.12.011

[22] Perez‐Gomez MV , Ortiz‐Arduan A , Lorenzo‐Sellares V , Vitamin D . Proteinuria: a critical review of molecular bases and clinical experience. Nefrologia. 2013;33:716‐726. 24089164 10.3265/Nefrologia.pre2013.Apr.12025

[23] Li YC , Kong J , Wei M , Chen ZF , Liu SQ , Cao LP . 1,25‐Dihydroxyvitamin D(3) is a negative endocrine regulator of the renin‐angiotensin system. J Clin Invest. 2002;110:229‐238. 12122115 10.1172/JCI15219 PMC151055

[24] Zhang Y , Kong J , Deb DK , Chang A , Li YC . Vitamin D receptor attenuates renal fibrosis by suppressing the renin‐angiotensin system. J Am Soc Nephrol. 2010;21:966‐973. 20378820 10.1681/ASN.2009080872 PMC2900963

[25] Cuppari L , Carvalho AB , Draibe SA . Vitamin D status of chronic kidney disease patients living in a sunny country. J Ren Nutr. 2008;18:408‐414. 18721735 10.1053/j.jrn.2008.05.004

[26] Isakova T , Gutiérrez OM , Patel NM , Andress DL , Wolf M , Levin A . Vitamin D deficiency, inflammation, and albuminuria in chronic kidney disease: complex interactions. J Ren Nutr. 2011;21:295‐302. 20817560 10.1053/j.jrn.2010.07.002

[27] Saha H . Calcium and vitamin D homeostasis in patients with heavy proteinuria. Clin Nephrol. 1994;41:290‐296. 8050209

[28] Stavroulopoulos A , Porter CJ , Roe SD , et al. Relationship between vitamin D status, parathyroid hormone levels and bone mineral density in patients with chronic kidney disease stages 3 and 4. Nephrol Ther. 2008;13:63‐67. 10.1111/j.1440-1797.2007.00860.x 18199106

[29] Kalkwarf HJ , Denburg MR , Strife CF , et al. Vitamin D deficiency is common in children and adolescents with chronic kidney disease. Kidney Int. 2012;81:690‐697. 22205356 10.1038/ki.2011.431 PMC3634332

[30] Freundlich M , Bourgoignie JJ , Zilleruelo G , et al. Bone modulating factors in nephrotic children with normal glomerular filtration rate. Pediatrics. 1985;76:280. 3839579

[31] Jankowska M , Szupryczyńska N , Dębska‐Ślizień A , et al. Dietary intake of vitamins in different options of treatment in chronic kidney disease: is there a deficiency? Transplantation Proc. 2016;48:1427‐1430. 10.1016/j.transproceed.2015.11.039 27496421

[32] Krassilnikova M , Ostrow K , Bader A , et al. Low dietary intake of vitamin D and vitamin D deficiency in hemodialysis patients. J Nephrol Ther. 2014;4:166. 25068077 10.4172/2161-0959.1000166 PMC4109326

[33] Young LR , Backus RC . Oral vitamin D supplementation at five times the recommended allowance marginally affects serum 25‐hydroxyvitamin D concentrations in dogs. J Nutr Science. 2016;5:e31. 10.1017/jns.2016.23 PMC4976120 27547394

[34] Klinger CJ , Hobi S , Johansen C , Koch HJ , Weber K , Mueller RS . Vitamin D shows in vivo efficacy in a placebo‐controlled, double‐blinded, randomised clinical trial on canine atopic dermatitis. Vet Rec. 2018;182:406. 29419484 10.1136/vr.104492

[35] Sharp CR , Selting KA , Ringold R . The effect of diet on serum 25‐hydroxyvitamin D concentrations in dogs. BMC Res Notes. 2015;8:442. 26374201 10.1186/s13104-015-1360-0 PMC4570747

[36] Wennogle SA , Priestnall SL , Suarez‐Bonnet A , et al. Comparison of clinical, clinicopathologic, and histologic variables in dogs with chronic inflammatory enteropathy and low or normal serum 25‐hydroxycholecalciferol concentrations. J Vet Intern Med. 2019;33:1995‐2004. 31496004 10.1111/jvim.15614 PMC6766529

[37] Michaud J , Naud J , Ouimet D , et al. Reduced hepatic synthesis of calcidiol in uremia. J Am Soc Nephrol. 2010;21:1488‐1497. 20595682 10.1681/ASN.2009080815 PMC3013518

[38] Chang Y‐M , Hadox E , Szladovits B , Garden OA . Serum biochemical phenotypes in the domestic dog. PLoS One. 2016;11:e0149650. 26919479 10.1371/journal.pone.0149650 PMC4769346

[39] Kaufmann M , Gallagher JC , Peacock M , et al. Clinical utility of simultaneous quantitation of 25‐hydroxyvitamin D and 24,25‐dihydroxyvitamin D by LC‐MS/MS involving derivatization with DMEQ‐TAD. J Clin Endocrinol Metab. 2014;99:2567‐2574. 24670084 10.1210/jc.2013-4388 PMC4079315

[40] Yousefzadeh P , Shapses SA , Wang X . Vitamin D binding protein impact on 25‐hydroxyvitamin D levels under different physiologic and pathologic conditions. Int J Endocrinol. 2014;2014:981581. 24868205 10.1155/2014/981581 PMC4020458

[41] Chesney RW . Interactions of vitamin D and the proximal tubule. Pediatr Nephrol. 2016;31:7‐14. 25618772 10.1007/s00467-015-3050-5

[42] Figuiredo‐Dias V , Cuppari L , Garcia‐Lopes MG , de Carvalho AB , Draibe SA , Kamimura MA . Risk factors for hypovitaminosis D in nondialyzed chronic kidney disease patients. J Ren Nutr. 2012;22:4‐11. 21652219 10.1053/j.jrn.2011.02.001

[43] Thrailkill KM , Jo C‐H , Cockrell GE , Moreau CS , Fowlkes JL . Enhanced excretion of vitamin D binding protein in type 1 diabetes: a role in vitamin D deficiency? J Clin Endocrinol Metab. 2011;96:142‐149. 20943786 10.1210/jc.2010-0980 PMC3038488

[44] Doorenbos CR , de Cuba MM , Vogt L , et al. Antiproteinuric treatment reduces urinary loss of vitamin D‐binding protein but does not affect vitamin D status in patients with chronic kidney disease. J Steroid Biochem. 2012;128:56‐61. 10.1016/j.jsbmb.2011.09.002 21958677

[45] Kalousova M , Dusilova‐Sulkova S , Zakiyanov O , et al. Vitamin D binding protein is not involved in vitamin D deficiency in patients with chronic kidney disease. Biomed Res Int. 2015;2015:492365. 26064917 10.1155/2015/492365 PMC4434169

[46] Barragry JM , France MW , Carter ND , et al. Vitamin‐D metabolism in nephrotic syndrome. Lancet. 1977;2:629‐632. 71448 10.1016/s0140-6736(77)92498-9

[47] Auwerx J , De Keyser L , Bouillon R , et al. Decreased free 1,25‐dihydroxycholecalciferol index in patients with the nephrotic syndrome. Nephron. 1986;42:231‐235. 3753749 10.1159/000183672

[48] Hamano T . Vitamin D and renal outcome: the fourth outcome of CKD‐MBD? Oshima award address 2015. Clin Exp Nephrol. 2018;22:249‐256. 29270765 10.1007/s10157-017-1517-3 PMC5838134

[49] Mirkovic K , Doorenbos CR , Dam WA , et al. Urinary vitamin D binding protein: a potential novel marker of renal interstitial inflammation and fibrosis. PLoS One. 2013;8:e55887. 23409077 10.1371/journal.pone.0055887 PMC3569442

[50] Chacar F , Kogika M , Sanches TR , et al. Urinary Tamm‐Horsfall protein, albumin, vitamin D‐binding protein, and retinol‐binding protein as early biomarkers of chronic kidney disease in dogs. Physiol Rep. 2017;5:e13262. 28576851 10.14814/phy2.13262 PMC5471429

[51] Lauridsen AL , Aarup M , Christensen AL , Jespersen B , Brixen K , Nexo E . Sensitive automated ELISA for measurement of vitamin D‐binding protein (Gc) in human urine. Clin Chem. 2005;51:1016‐1018. 15914783 10.1373/clinchem.2004.045658

[52] Yildirim I , Hur E , Kokturk F . Inflammatory markers: C‐reactive protein, erythrocyte sedimentation rate, and leukocyte count in vitamin D deficient patients with and without chronic kidney disease. Int J Endocrinol 2013;2013:802165, 1, 6. 10.1155/2013/802165 PMC3710598 23878538

[53] Littman MP . Protein‐losing nephropathy in small animals. Vet Clin N Am‐Small. 2011;41:31‐62. 10.1016/j.cvsm.2010.09.006 21251510

[54] Throop JLKM , Cohn LA . Albumin in health and disease: causes and treatment of hypoalbuminemia. Compendium. 2004;26:940‐949.

[55] Cray C , Zaias J , Altman NH . Acute phase response in animals: a review. Comp Med. 2009;59:517‐526. 20034426 PMC2798837

[56] Gow AG , Else R , Evans H , Berry JL , Herrtage ME , Mellanby RJ . Hypovitaminosis D in dogs with inflammatory bowel disease and hypoalbuminaemia. J Small Anim Pract. 2011;52:411‐418. 21797872 10.1111/j.1748-5827.2011.01082.x

[57] Baeke F , Takiishi T , Korf H , Gysemans C , Mathieu C . Vitamin D: modulator of the immune system. Curr Opin Pharmacol. 2010;10:482‐496. 20427238 10.1016/j.coph.2010.04.001

[58] Mangin M , Sinha R , Fincher K . Inflammation and vitamin D: the infection connection. Inflamm Res. 2014;63:803‐819. 25048990 10.1007/s00011-014-0755-z PMC4160567

[59] Yonemura K , Fujimoto T , Fujigaki Y , Hishida A . Vitamin D deficiency is implicated in reduced serum albumin concentrations in patients with end‐stage renal disease. Am J Kid Dis. 2000;36:337‐344. 10922312 10.1053/ajkd.2000.8984

[60] Nabity MB , Boggess MM , Kashtan CE , Lees GE . Day‐to‐day variation of the urine protein: creatinine ratio in female dogs with stable glomerular proteinuria caused by X‐linked hereditary nephropathy. J Vet Intern Med. 2007;21:425‐430. 17552446 10.1892/0891-6640(2007)21[425:dvotup]2.0.co;2

[61] Shropshire S , Quimby J , Cerda R . Comparison of single, averaged, and pooled urine protein:creatinine ratios in proteinuric dogs undergoing medical treatment. J Vet Intern Med. 2018;32:288‐294. 29171088 10.1111/jvim.14872 PMC5787151

[62] Major JM , Graubard BI , Dodd KW , et al. Variability and reproducibility of circulating vitamin D in a nationwide U.S. population. J Clin Endocrinol Metab. 2013;98:97‐104. 23144464 10.1210/jc.2012-2643 PMC3537087

[63] Brescia VTM , Cardinali R . Biological variability of serum 25‐hydroxyvitamin D and other biomarkers in healthy subjects. Lab Med. 2013;44:20‐24.

[64] Susantitaphong P , Nakwan S , Peerapornratana S , et al. A double‐blind, randomized, placebo‐controlled trial of combined calcitriol and ergocalciferol versus ergocalciferol alone in chronic kidney disease with proteinuria. BMC Nephrol. 2017;18:19. 28088187 10.1186/s12882-017-0436-6 PMC5237567

[65] Wu CC , Liao MT , Hsiao PJ , et al. Antiproteinuria effect of calcitriol in patients with chronic kidney disease and vitamin D deficiency: a randomized controlled study. J Ren Nutr. 2020;30:200‐207. 31704188 10.1053/j.jrn.2019.09.001

