# Supplementary material for: Association between vitamin D metabolites, vitamin D binding protein, and proteinuria in dogs
Source: J Vet Intern Med. 2020 Oct 7;34(6):2468–77. doi: 10.1111/jvim.15912 (PMC7694856; doi:10.1111/jvim.15912)
Supplement: Supplementary file 1 — Appendix S1: Supporting Information [file JVIM-34-2468-s001.pdf]

1. Diagnostic plots for multivariable regression models associated with Table 4.

A. **1,25(OH)$_2$D**

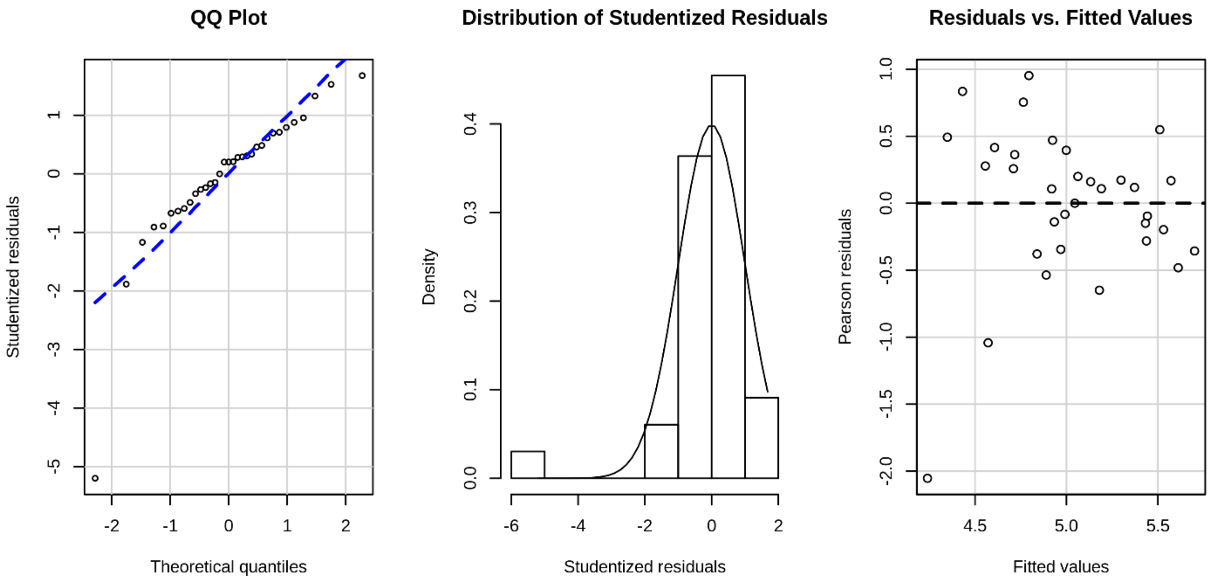

B. **24,25(OH)$_2$D**

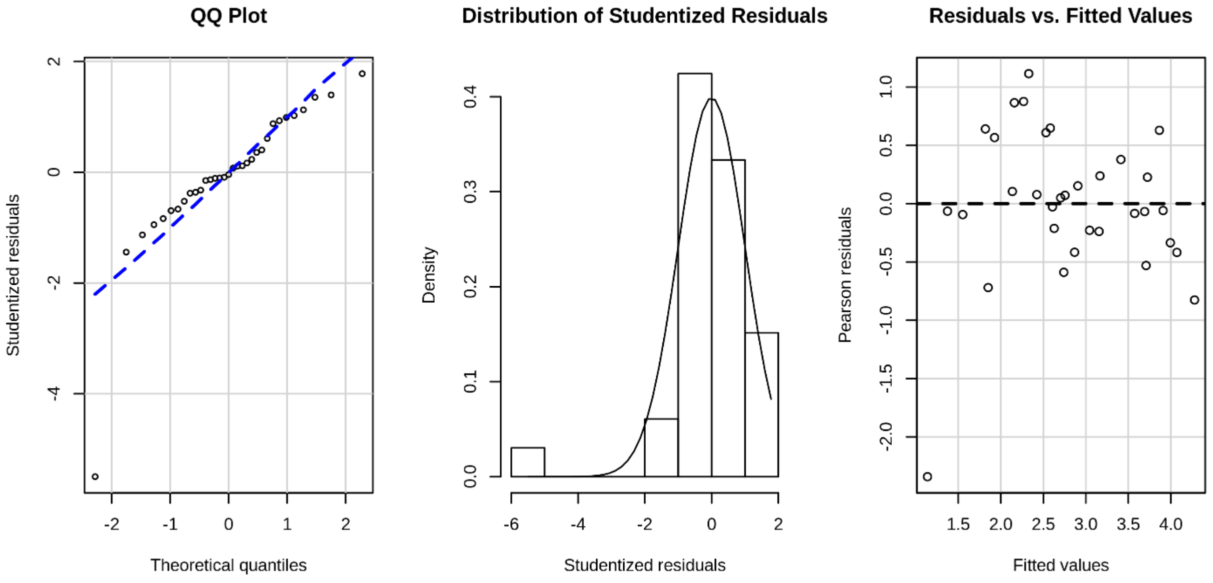

C. **25(OH)D**

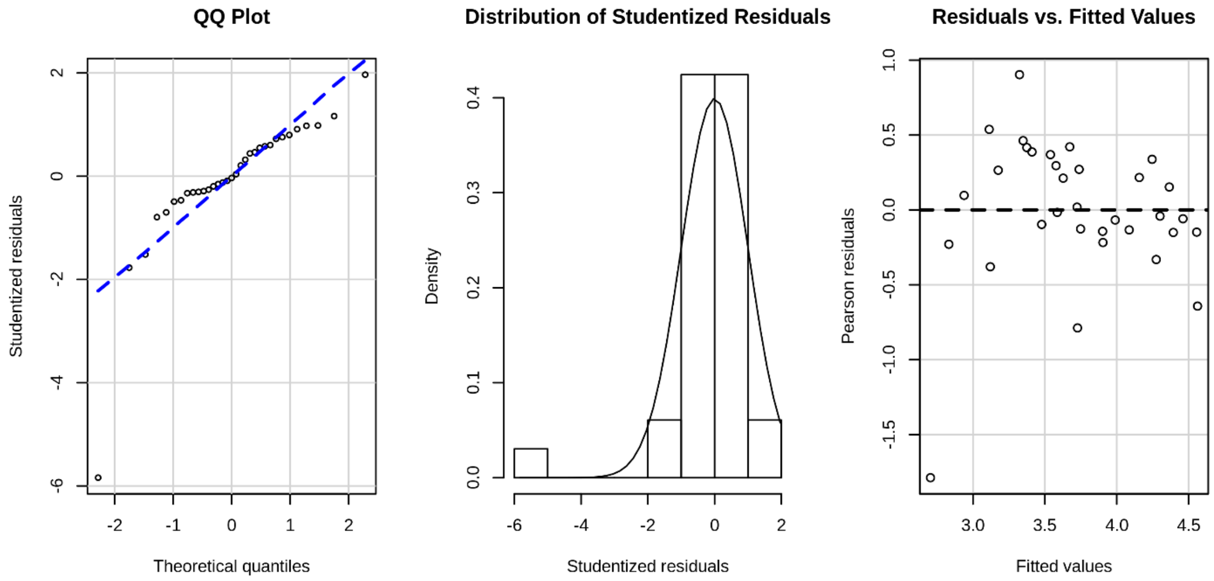

2. Diagnostic plots for multivariable regression models associated with Table 5.

A. **1,25$(OH)_2$D**

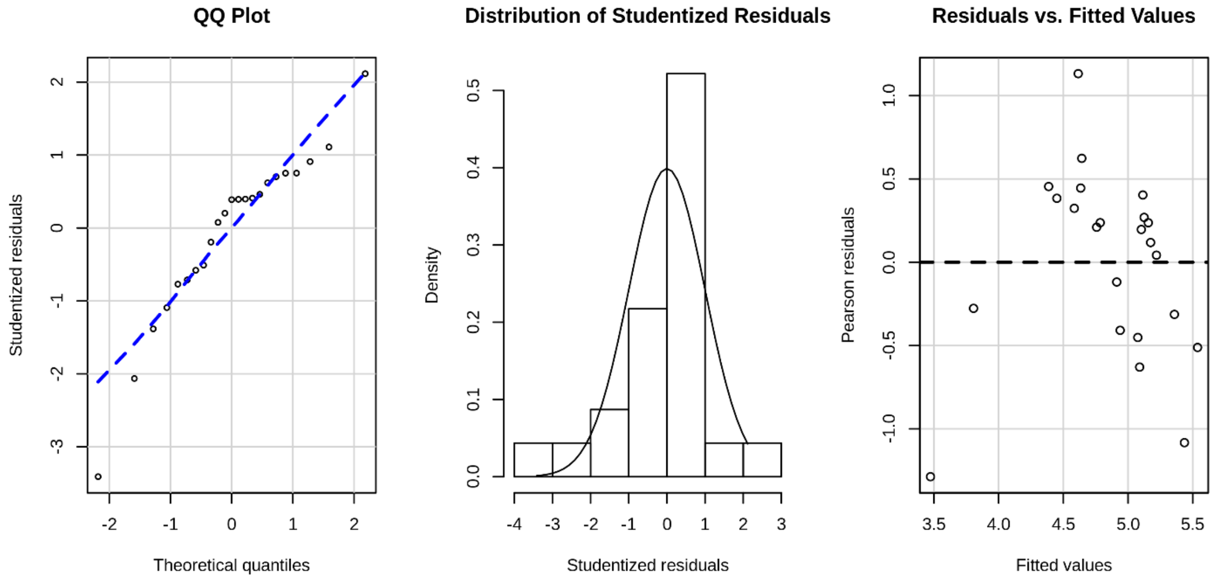

B. **24,25$(OH)_2$D**

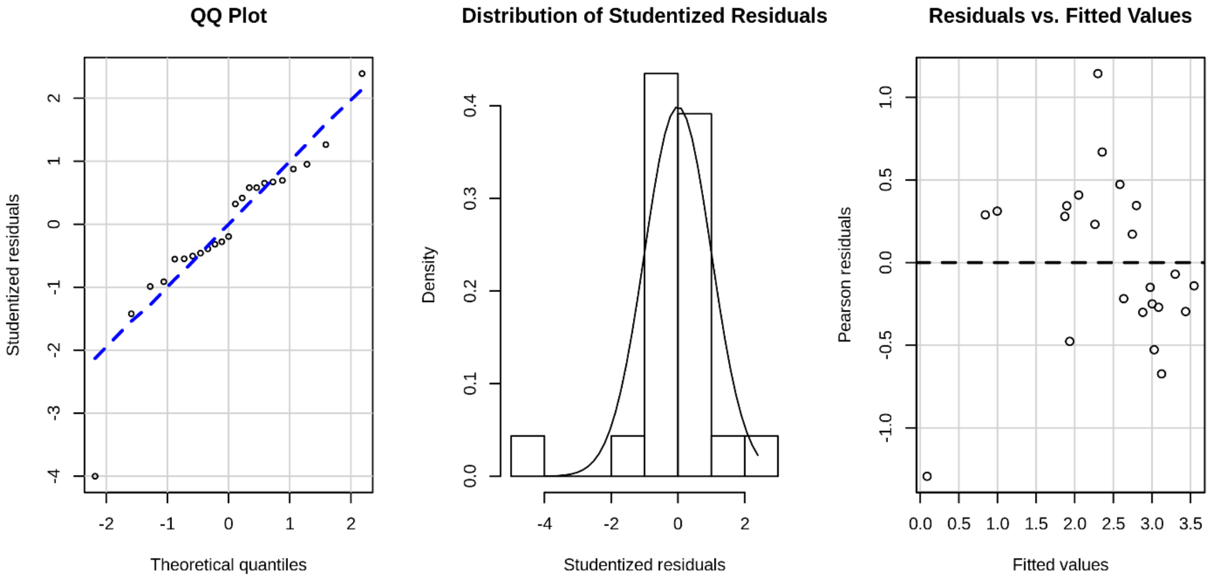
QQ Plot
Studentized residuals
Theoretical quantiles
Distribution of Studentized Residuals
Density
Studentized residuals
Residuals vs. Fitted Values
Pearson residuals
Fitted values

C. **25(OH)D**

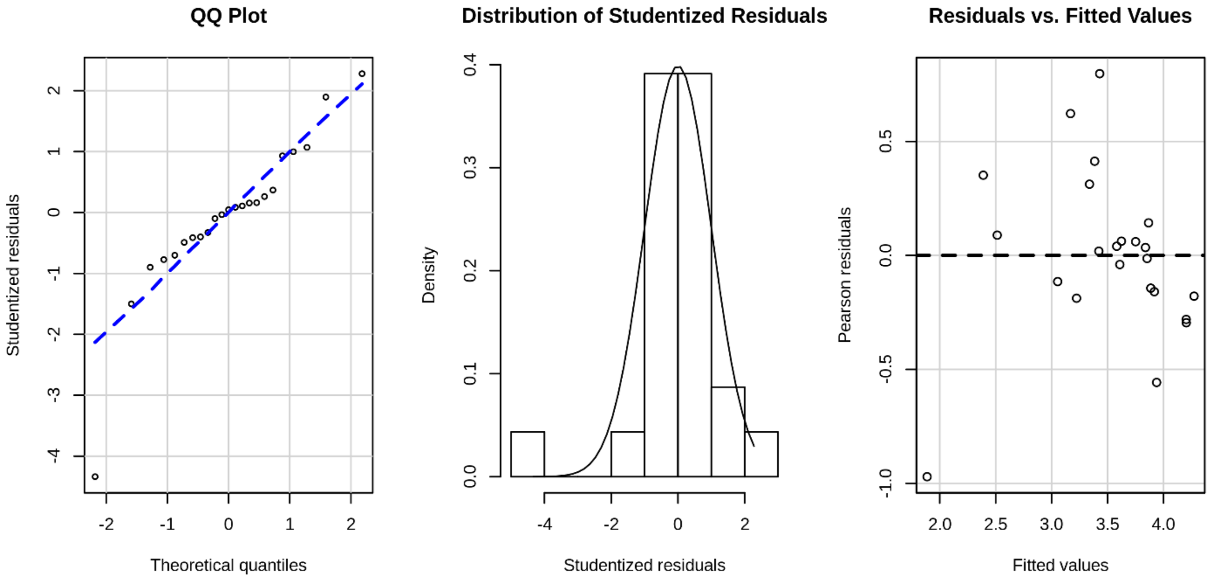
QQ Plot
Studentized residuals
Theoretical quantiles
Distribution of Studentized Residuals
Density
Studentized residuals
Residuals vs. Fitted Values
Pearson residuals
Fitted values
